# Les raisons du refus et abandon de soins aux urgences chirurgicales du Centre Hospitalier et Universitaire de Bouaké, Côte d’Ivoire

**DOI:** 10.11604/pamj.2021.38.291.22340

**Published:** 2021-03-19

**Authors:** Loukou Blaise Yao, Achié Jean Régis Akobe, Kouamé Innocent M’Bra, Bada Justin Léopold Niaoré Sery, Kouamé Jean-Eric Kouassi, Aya Adelaïde Natacha Kouassi, Yao Aboh Ganin Robert Arnaud Assere, Koffi Léoplod Krah, Michel Kodo

**Affiliations:** 1 Service d’Orthopédie-Traumatologie du Centre Hospitalier Universitaire de Bouaké, Bouaké 01, Côte d’Ivoire

**Keywords:** Abandon de soins, médecine traditionnelle, moyen financier, traumatologie, Abandonment of care, traditional medicine, financial resources, traumatology

## Abstract

Le refus et l´abandon de soins est un comportement fréquemment observé dans notre pratique quotidienne. L´objectif de cette étude était de décrire l´épidémiologie et d´identifier les raisons de ces refus et abandons de soins. Une étude prospective a été réalisée du 1^er^ janvier 2018 au 31 décembre 2018 aux urgences chirurgicales du Centre Hospitalier et Universitaire de Bouaké. Elle a concerné tous les patients admis pour des lésions traumatiques et ayant refusé ou abandonné les soins médicaux. Il a été colligé 106 cas de refus et abandon de soins sur 662 patients admis pour des pathologies traumatiques des membres pendant cette période donc une fréquence de 16%. L´âge moyen était de 37 ans. Il y avait 77 hommes (72,6%). Les patients exerçaient dans le secteur tertiaire dans 56,6% (n= 60). Les lésions observées étaient dominées par les fractures fermées 82,1% (n= 87) et les membres pelviens étaient les plus atteints 78,3% (n=83). Le traitement proposé était chirurgical (n=85; 80,2%) et orthopédique (n=21; 19,8%). Le coût du traitement orthopédique était estimé à 26 500 FCFA (40 euros) et à 250 000 FCFA (380 euros) pour le traitement chirurgical. Ces coûts variaient en fonction de l´implant prescrit et de la localisation. Le refus des soins était demandé par le patient lui-même (n=30; 28,3%), et par les parents (n=76; 71,7%). Les motifs retrouvés étaient dominés par le problème financier (n=62; 58,5%), la confiance en la médecine traditionnelle (n=42; 39,6%), la croyance religieuse (n=2; 1,9%). Le délai moyen de refus de soins était de 22 heures. Il a été noté 88,7% (n=94) de décharge signées et 11,3% (n=12) d´évasion. Le refus des soins est récurrent dans notre contexte et est lié à l´insuffisance du système de santé en termes de prise en charge des patients à ressource financière limitée.

## Abstract (en)

The refusal and abandonment of treatment is a behavior frequently observed in our daily practice. The purpose of this study was to describe the epidemiology and to identify the reasons for refusals and abandonment of treatment. We conducted a prospective study in the emergency surgery at the University Hospital Center of Bouake from 1^st^ January 2018 to 31^st^ December 2018. It involved all patients admitted with traumatic lesions who had refused or abandoned treatment. Data from 106 cases (16%) of refusal and abandonment of treatment out of 662 cases admitted with limb traumas were examined over this period. The average age of patients was 37 years. The study enrolled 77 men (72.6%). Tertiary sector workers accounted for 56.6% (n= 60) of cases. Lesions were dominated by closed fractures (82.1%; n= 87) and pelvic limbs were the most achieved (78.3%; n=83). Treatment was based on surgery (n=85; 80.2% ) and orthopaedic treatment (n=21; 19.8%). The cost of orthopedic treatment was estimated at 26 500 CFA francs (40 euros) while at 250 000 FCFA (380 euros) for surgical treatment. These costs varied as a function of implant prescribed and its location. Refusal of tratment was expressed by patients (n=30; 28.3%) and by parents (n=76; 71.7%). Reported reasons were dominated by financial problems (n=62; 58.5%), trust in traditional medicine (n=42; 39.6%), religious belief (n=2; 1.9%). The average time of refusal was 22 hours. Eighty eight point seven percent (n=94) of patients signed discharge while 11.3% (n=12) escaped. Refusal of care is a recurrent theme in our context and is due to inadequate health care management of people with limited financial resources.

## Introduction

L´accès aux soins de santé est un enjeu important dans les pays en voie de développement par rapport aux pays développés où un système de couverture maladie universelle et sociale est mis en place, facilitant le recours aux soins de santé [1]. Le consentement de la personne examinée ou soignée est recherché dans tous les cas et répond à la loi relative au droit des malades [2]. Le refus et l´abandon de soins est une décision volontaire plus ou moins consciente considérée, comme une difficulté d´accès à des soins, et peut survenir à tout moment d´un itinéraire de soins [3]. Plusieurs formes d´expression de refus (orale, écrite, autres alternatives, peur et découragement) par le patient ou les parents sont retrouvées [4]. Le refus et l´abandon de soins est devenu récurrent dans le service et plusieurs facteurs seraient incriminés. L´objectif de cette étude était de décrire l´épidémiologie et d´identifier les raisons du refus et abandon de soins.

## Méthodes

**Type et lieu de l´étude:** il s´agissait d´une étude prospective descriptive réalisée du 1^er^ Janvier 2018 au 31 Décembre 2018 aux urgences chirurgicales du Centre Hospitalier et Universitaire (CHU) de Bouaké en Côte d´Ivoire.

**Population d´étude:** cette étude a concerné tous les patients admis pour une lésion traumatique des membres ayant refusé ou abandonné les soins après le diagnostic et la décision thérapeutique. Les patients ayant demandé un transfert pour la poursuite des soins dans une autre institution n´ont pas été inclus dans l´étude.

**Collecte des données:** le diagnostic des lésions a été fait à partir de l´anamnèse, l´examen clinique et les radiographies standards des membres. Lorsque la décision du refus de soins est constatée, un interrogatoire est mené au près du patient et des parents pour recueillir les raisons. Les informations concernant la fréquence, l´âge, le sexe, la profession, l´ethnie, la religion, l´étiologie, les lésions, le traitement proposé avec son coût et les raisons de l´abandon des soins ont été recherchées.

**Considérations éthiques:** les informations ont été recueillies après consentement éclairé des patients et avec l´accord de la direction médicale scientifique du CHU de Bouaké.

## Résultats

**Données socio démographiques:** il a été colligé 106 cas de refus et abandon de soins sur 662 patients admis pour une pathologie traumatique des membres pendant cette période donc une fréquence de 16%. Il y avait 77 hommes (72,6%) et 29 femmes (27,4%). L´âge moyen était de 37 ans (4-88). Les patients étaient musulmans (n=51; 48,1%), chrétiens (n=40; 37,7%) et animistes (n=15; 14,2%). Les patients étaient d´ethnie Sénoufo (n=55; 51,8%), Baoulé (n=43; 40,6%), Bété (n=6; 5,7%) et Gouro (n=2; 1,9%). La profession des patients est résumée dans le Tableau 1.

**Tableau 1:** répartition de la profession

Profession	Nombre (n=106)	Pourcentage (%)
Cultivateur	27	25,5
Elève	23	21,7
Commerçant	20	18,9
Ménagère	12	11,3
Chauffeur	10	9,4
Fonctionnaire	6	5,7
Couturier	3	2,8
Sans emploie	3	2,8
Homme religieux	2	1,9

**Données étiologiques, lésionnelles et thérapeutiques:** les étiologies et les lésions observées des membres sont répertoriées dans le Tableau 2. Les accidents de la voie publique impliquaient les motocycles dans 77 cas (72,1%). Les lésions siégeaient aux membres pelviens (n=83; 78,3%) et aux membres thoraciques (n=23; 21,7%). Le traitement était chirurgical (n=85; 80,2%) et orthopédique (n=21; 19,8%). Le coût pour le traitement orthopédique était estimé à 26 500 FCFA (40 euros) et à 250 000 FCFA (380 euros) pour le traitement chirurgical. Ces coûts variaient en fonction de l´implant prescrit et de la localisation.

**Tableau 2:** répartition des étiologies et des lésions observées

Données	Nombre (n=106)	Pourcentage (%)
Etiologies		
Accident de la voie publique	80	75,5
Accident de travail	15	14,2
Accident de sport	10	9,4
Agression par arme à feu	1	0,9
**Lésions observées**		
Luxation d´épaule	3	2,8
FF humérus	5	4,7
Coude flottant	1	0,9
FF 2 os avant-bras	14	13,2
FF poignet	3	2,8
FF fémur	29	27,4
FF plateau tibial	4	3,7
FO plateau tibial	1	0,9
FF jambe	22	20,8
FO jambe	11	10,5
FF pilon tibial	3	2,8
FF du pied	10	9,5

* FF= fracture fermée; *FO = fracture ouverte

**Données sur le refus de soins:** le refus de soins a été demandé dans un délai moyen de 22 heures (3 heures et 7 jours). La demande du refus a été effectuée par les parents du patient (n=72; 71,7%) et par le patient lui-même (n=30; 28,3%). Les raisons du refus de soins sont répertoriées dans le Tableau 3. Les parents ont signé une décharge écrite dans 19,8% (n=21), les patients dans 68,9% (n=73) et 11,3% (n=12) d´évasion ont été notés.

**Tableau 3:** répartition des raisons du refus de soins

Raisons du refus	Nombre (n=106)	Pourcentage (%)
Problèmes financiers	62	58,5
Croyance au traitement traditionnel	42	39,6
Croyance religieuse	2	1,9

## Discussion

Cette étude a révélé qu´il s´agissait d´une attitude fréquente dans le service. Il s´agissait de sujets jeunes de sexe masculin, présentant des lésions fermées touchant les membres pelviens majoritairement. Plusieurs raisons ont été identifiées dont le problème financier comme raison fondamentale, la croyance au traitement traditionnel et la croyance religieuse. La prise en charge des patients dans les pays en voie de développements est difficile devant le système de santé et le plateau technique limités [5,6]. Le phénomène du refus et abandon de soins est fréquent en Afrique subsaharienne [7,8]. Les patients étaient des adultes jeunes majoritairement de sexe masculin [9]. Cette tranche constitue la population active et mobile victime d´accident de la voie publique [10]. Il s´agissait donc de patients jeunes pleinement responsables, capables de comprendre les propositions thérapeutiques et les conséquences du refus [2]. Les Sénoufos et les Baoulés prédominaient, il s´agissait des populations du Centre et du Nord du pays, connues pour leurs fortes croyances au traitement traditionnel ainsi que leurs recours aux pratiques rebouteuses. Les soins et leurs coûts (bilans médicaux, implants et hospitalisation) sont à la charge des patients qui n´ont aucune couverture sociale avec un revenu faible. Les problèmes financiers sont retrouvés comme facteur principal des difficultés de prise en charge dans les pays en voie de développement [11,12]. Dans cette présente étude la majorité des patients exerçaient dans le secteur tertiaire avec un faible revenu.

Les problèmes financiers limitent l´accessibilité des patients aux hôpitaux et sont vus comme une barrière d´accès aux soins de santé [12,13]. Les difficultés financières sont également retrouvées dans certains pays développés malgré le système de couverture maladie mis en place [13]. La croyance au traitement traditionnel a été retrouvée comme facteur potentiel d´abandon dans cette série. La promotion par les médias et l´encadrement de la médecine traditionnelle a connu un essor dans le pays. Ces raisons incitent donc cette population à forte croyance culturelle à un libre accès à cette médecine. Aussi une mauvaise expérience vécue par un membre de l´entourage du patient dans un centre de santé influençait les patients aux refus immédiats des soins [14]. Les lésions observées étaient plus les fractures fermées des membres. Elles constituaient une raison car selon les patients ces lésions n´étaient pas assez graves à traiter par le traitement traditionnel. Il demeure un fatalisme culturel selon lequel les guérisseurs traitent mieux les fractures que les médecins orthodoxes [7,15]. La croyance religieuse représentait aussi un facteur du refus. Les patients l´exprimaient par leur foi au créateur en ces termes «DIEU me guérira !».

La majorité des patients ont signé une décharge après avoir refusé les soins malgré les informations sur les conséquences du refus. Par ailleurs même si la décharge signée n´a aucune valeur légale [16,17], elle constituait une preuve démontrant que les informations sur les conséquences du refus étaient bien expliquées aux patients et aux parents. En outre, le refus et l´abandon sont pensés comme des choix. Ce sont néanmoins parfois des défaillances du système de santé qui amènent les personnes à se tourner vers d´autres formes de recours, dont l´efficacité n´est pas nécessairement prouvée mais qui offrirait un traitement à moindre coût, une prise en compte des besoins et des attentes.

## Conclusion

Cette étude a montré que le refus et abandon des soins représente une attitude fréquente dans le service. Plusieurs raisons ont été identifiées dont le manque de moyen financier comme raison fondamentale, la croyance au traitement traditionnel et la croyance religieuse. La mise en place effective de l´Assurance Maladie Universelle (AMU) et d´un système de tarification décroissante dans les centres de santé pourrait limiter ces abandons de soins et assurer au mieux la santé de la collectivité.

### Etat des connaissances sur le sujet

- Ce phénomène est observé et documenté dans d´autres structures sanitaires;
- La croyance culturelle fréquente aux pratiques rebouteuses pour le traitement des fractures;
- L´absence de système de couverture sociale de soins de santé.

### Contribution de notre étude à la connaissance

- La fréquence de ce phénomène aux urgences chirurgicales du CHU de Bouaké est de 16%;
- les raisons de ce phénomène dans notre contexte régional sont le manque de moyen financier comme raison fondamentale, la croyance au traitement traditionnel et la croyance religieuse.
